# Nunataks or massif de refuge? A phylogeographic study of *Rhodiola crenulata* (Crassulaceae) on the world’s highest sky islands

**DOI:** 10.1186/s12862-018-1270-6

**Published:** 2018-10-16

**Authors:** Yuan-Zhen Zhang, Ruo-Wei Zhu, Da-Lv Zhong, Jian-Qiang Zhang

**Affiliations:** College of Life Sciences, Shaanxi Normal University, No. 620, West Chang’an Avenue, Chang’an District, Xi’an, 710119 China

**Keywords:** Nunataks, Massif de refuge, Phylogeography, Qinghai-Tibetan plateau, Quaternary climatic oscillations, Quaternary speciation

## Abstract

**Background:**

Quaternary climatic oscillations had tremendous effects on the current distribution of species. Here, we aim to elucidate the glacial history of *Rhodiola crenulata*, a perennial herb almost exclusively restricted to rock crevices on mountain peaks, and to test whether the nunatak or massif de refuge hypotheses could explain its distribution pattern.

**Results:**

Six haplotypes and six ribotypes were detected in the cpDNA data set and the ITS data set, respectively. The divergence of *R. crenulata* and its closest relatives was dated have occurred ca. 0.65 Mya, during the Naynayxungla glaciation on the QTP. Mismatch distribution analysis suggested that the species experienced a range expansion around 0.31 Mya. Populations with high genetic and haplotype diversity were found on the QTP platform as well in the Hengduan Mountains. The ecological niche modeling results showed that there were suitable habitats on both the QTP platform and in the Hengduan Mountains during the LGM.

**Conclusion:**

Our results support a scenario that both nunataks and the massif de refuge hypotheses could explain the distribution of *R. crenulata*. We also confirmed that Quaternary climatic oscillations could promote plant speciation in some circumstances. This study adds to a growing body of evidence suggesting that the QTP plant lineages exhibited diverse reactions to the Quaternary climatic oscillations.

**Electronic supplementary material:**

The online version of this article (10.1186/s12862-018-1270-6) contains supplementary material, which is available to authorized users.

## Background

Mountains have long been recognized as island-like systems [1–3]. These so-called “sky islands” could provide us with important insights into evolutionary mechanisms of island systems. Limited size, distinct boundaries, isolation and dispersal limitation are shared attributes of these systems. Researchers since Darwin have documented the divergence of populations in relation to allopatry, evolutionary modification of form and function, and strikingly rapid diversification on island systems, including sky islands both on the Qinghai-Tibetan Plateau (QTP) and in the Andes [4]. However, how plants on such sky islands responded to the Quaternary climate oscillations, which had tremendous effects on species ranges, has not been fully explored.

The current distribution of genetic lineages of species in the Northern Hemisphere was greatly influenced by the Quaternary climatic oscillation [5, 6]. During the ice ages, the decreasing temperature and reduced altitude of the mountain snow line [7] allowed organisms originally living in alpine zones either to move to lower elevations or to find a suitable refugium in situ. Two hypotheses exist relating to how alpine organisms survive glacial periods based on studies in the Alps and the north Atlantic area [8–10]: the “nunatak” hypothesis suggests survival on mountain tops within the ice sheet [11], whilst the massif de refuge hypothesis suggests that the periphery of mountain ranges provided large refugia for alpine species, allowing the refugees to re-occupy alpine zones after the retreat of glaciers [12]. Previous phylogeographic studies conducted on the QTP, one of the largest and highest plateaus in the world, provide us with important insights into how plants on the plateau reacted to the Pleistocene climate oscillation. Three patterns have been revealed: contraction to the Hengduan Mountains during glaciations and subsequent recolonization onto the plateau [13, 14], which corresponds to the massif de refuge hypothesis; surviving in refugia that existed on the plateau platform during glaciations and local expansion during interglacial or postglacial [15, 16]; and the “microrefugia” hypothesis (i.e., multiple microrefugia during the Last Glacial Maximum (LGM, ca. 20 kya) across the whole of the current distribution range of species), which was confirmed by two previous studies of woody plants on the QTP [17, 18]. Although such studies shed important light on the evolutionary history of alpine species on the QTP during the glacial period, most of them have focused on woody plants or herbs of alpine meadows. Few studies have been conducted to illustrate how species growing in rock crevices on mountain peaks reacted to the Quaternary climatic oscillations.

**Fig. 1:**
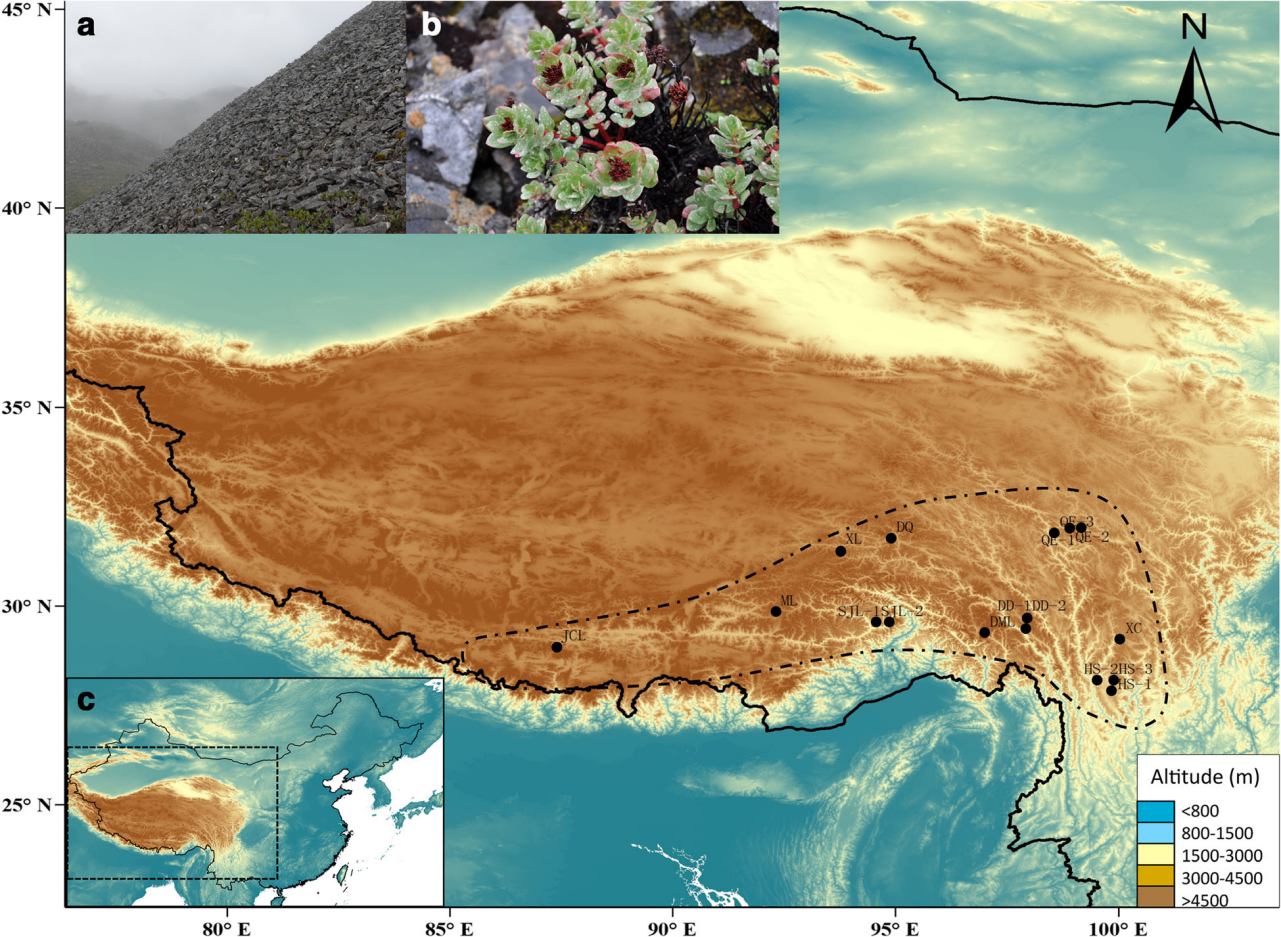
Distribution, habitat and morphology of *R. crenulata*. **a** A photograph of the habitat of *R. crenulata*; **b** plants in fruit; **c** presumed distribution range of the species and sampling location of the 16 populations in the study. Presumed distribution range of this species according to herbarium records is indicated by black dashed lines. Our sampling covered the main part of the distribution area of this species

*Rhodiola crenulata* (HK. f. et Thoms.) H. Ohba, our study species, is almost exclusively restricted to rock crevices on mountain peaks (Fig. 1), from 4300 m to 5600 m in elevation [19], which makes it one of the highest living vascular plants on the QTP. Such a distribution pattern represents a classical continental island system, with mountain peaks regarded as isolated islands. The species’ affinity for high alpine and nival altitudes suggests that it would survive on nunataks, which certainly offered extremely harsh conditions for survival. Consequently, our first goal was to elucidate the glacial history of *R. crenulata*, and to test the nunatak and massif de refuge hypotheses. *Rhodiola crenulata* has been used as an important source of adaptogens, hemostatics, and tonics in traditional Tibetan medicines for thousands of years [20]. The accelerated and uncontrolled exploitation of *R. crenulata* has made it extremely endangered. Thus our second goal was to explore the genetic diversity and distribution pattern of this species to offer more information for conservation and management programs. To achieve our goals, we examined plastid and nuclear ITS markers and used ecological niche models (ENMs) to conduct a phylogeographic study. The ENMs that are widely used in phylogeographic studies, can provide important evolutionary and biological insights by allowing the evaluation of biogeographic hypotheses or to interpret genetic diversity patterns [21, 22].

## Methods

### Population sampling

Our sampling did not require specific permits for the locations involved as we held a permission letter from College of Life Sciences, Shaanxi Normal University, Xi’an relating to colleting samples. We sampled 16 populations across the distribution range of *R. crenulata* during the summers of 2016 to 2017. For each population, we generally sampled 10–20 individuals. Each sampled individual in every population was at least 20 m away from any other to avoid sampling clonal individuals. In total, we collected 253 individuals and dried their leaves with silica gel. Based on a previous phylogenetic study, we chose one individual of *R. wallichiana* (Hk.) S. H. Fu to act as an outgroup [23].

### DNA extraction, PCR amplification, cloning and sequencing

We used a Plant Genomic DNA Kit (TianGen Biotech, Beijing, China) to extract DNA from silica-gel dried leaves. PCR amplification was conducted in a 20 μl system (2 μl 10 × buffer, 0.5 μl of each primer, 0.4 μl of dNTP mixture, 1 U of Taq polymerase, 1 μl template genomic DNA), using primers for ITS, ITS-1 and ITS-4 [24], for *trnL-F*, c and f [25] and for *trnS-G*, trnS and trnG [26]. We used the PCR conditions described by Liu et al. [27]. For chloroplast sequences and most of the ITS sequences, direct sequencing was conducted. For those ITS sequences with multiple peaks, we ligated the PCR product into pGEM-T Easy Vector using a Promega Kit (Promega Corporation, Madison, WI, USA), and chose a plasmid containing the right insertion to sequence. We conducted all sequencing on a 3730 automatic DNA sequencer at Tsingke Biotech, Xi’an, China. The contigs obtained were edited and assembled using ContigExpress (a component of Vector NTI Suite 6.0, InforMax). ClustalW v. 1.7 [28] was used to align the data set, and BioEdit v. 7.0.1. was used to check the aligned sequence by eye.

### Phylogenetic analysis and divergence time inference

Sequences from two cpDNA regions (*trn*L-F and *trn*S-G) were concatenated into a matrix because the chloroplast genome can be considered to have evolved as a single entity in plants. Segregating sites and haplotypes were extracted using DnaSP v5 [29]. To infer phylogenetic relationships between extracted haplotypes/ribotypes, we applied the Maximum Parsimony method in PAUP* v. 4.0b10 [30] and then conducted Bayesian inference analysis. We specified the following parameters for the parsimony calculation: heuristic searches with MULTREES, TBR branch swapping, 1000 replicates of random addition sequences for starting trees. Bootstrap values [31] were obtained from a run of 1000 replicates, with ten replicates of random addition sequences and NNI branch swapping.

Because Bayesian inference requires a nucleotide substitution model, we determined one for each data set with the Akaike information criterion (AIC) in Modeltest v. 3.7 [32, 33] (TPM2uf + G for the plastid data set, and SYM + G for the ITS data set). MrBayes v. 3.2.1 (nst = 6, rates = gamma for the plastid data set, nst = 6, rates = gamma for the ITS data set) [34] was used the inference. We surveyed 10,000,000 generations of the four Metropolis-coupled Markov chains, and sampled every 1000 generations. The average stand deviation of split frequencies was used to assess the convergence of two independent runs, and the threshold was set to 0.05. We discarded the first 25% of generations as burn-in, and constructed a 50%-majority rule consensus tree with the remainder. The posterior proportion for each node was used to estimate robustness of the BI trees. In addition, we constructed haplotype/ribotype networks with NETWORK v. 4.2.0.1 [35] to reveal relationships between sequences with shallow genetic divergences.

To infer the divergence time between lineages, we first examined the molecular clock hypothesis with a likelihood ratio test in PAUP* v. 4.0b10. The results showed that both the ITS and cpDNA data sets fit the molecular clock hypothesis (2logeLR = 1.36, df = 5, *p* > 0.05 for the ITS data, and 2logeLR = 0.78, df = 5, *p* > 0.05 for the plastid data). We then used both the ITS and plastid data sets to conduct a dating analysis using the BEAST software [36]. All parameters were sampled every 1000 generations from 10,000,000 MCMC generations with the first 25% as burn-in. Tracer [36] was used to examine the convergence of chains. As far as we know, there are no reliable fossils of *R. crenulata* and its relatives available; we therefore used substitution rates to estimate divergence times. It has been reported that the substitution rate of nrITS in shrubs and herbs varies from 3.46 × 10^− 9^ to 8.69 × 10^− 9^ s/s/y [37], thus we used a normal distribution prior to cover these ranges with a 95% confidence interval (6.075 × 10^− 9^ 1.590 × 10^− 9^). The evolutionary rate of the plastid markers was assumed to be 2 × 10^− 9^ s/s/y [38].

### Population genetics

We used DnaSP v5 [29] to estimate the population genetic diversity indexes: haplotype diversity (*h*) and nucleotide diversity (*π*) for each population (*h*_S_*, π*_S_) and at the species level (*h*_T_*, π*_T_). We also calculated average gene diversity within the population (*H*_S_), total gene diversity (*H*_T_) and between population divergence (*G*_ST_, *N*_ST_) using the program PERMUT (available at https://www6.bordeaux-aquitaine.inra.fr/biogeco/Production-scientifique/Logiciels/Contrib-Permut/Permut) with 1000 permutation tests. If the *N*_ST_ value is larger than *G*_ST_, it means that closely related haplotypes will appear in the same area with higher probability than remotely related ones, thus indicating the presence of phylogeographic structure [39]. The Mantel test with 1000 random permutations was implemented to test the significance of isolation by distance between populations using ARLEQUIN v. 3.5 [40].

To see if there is any spatial structure in populations of *R. crenulata*, we used SAMOVA v. 1.0 [41] to assess the spatial genetic pattern. By maximizing the *F*_CT_ value, the program can search different *K* values that the user defines as groups of geographically adjacent populations. We ran the program repeatedly with *K* values from 2 to 10. After defining geographic groups, we calculated the amount of variation among populations within a geographic group and within a population using the hierarchical analysis of molecular variance (AMOVA) framework, which was run using ARLEQUIN v. 3.5 [40]. We used a nonparametric permutation procedure with 1000 permutations to test for any significant difference.

Then we inferred signatures of demographic expansion by calculating Tajima’s *D* and Fu’s *F*s statistics [42, 43] and conducting a mismatch distribution analysis [44, 45]. *D* and *F*s statistics are significantly negative if there is population expansion, as an excess of rare new mutations will originate during the expansion process. A previous study has shown that mismatch distribution is not affected by population structure [46], thus we pooled haplotypes of each clade together to conduct the analysis. The difference between observed mismatch distributions and expected distribution under a sudden expansion model [44, 47] was tested by examining the sum of squared deviations (*SSD*) and the raggedness index (*HRag*) [48] with 1000 parametric bootstrap replicates. If expansion was detected in a group, the parameter-value *τ* was used to estimate the expansion time (in generations) with the equation *t* = *τ*/2*u* [44, 46], where *u* = *μ* × *k* × *g*, in which μ represents substitution rate (s/s/y), *k* represents the length of aligned matrix, and *g* is the generation time in years. For the present study, *k* was 1531 bp, and the substitution rate for the cpDNA genome was assumed to be 2 × 10^− 9^ s/s/y [38, 49]. Generation time was estimated to be 10 years based on personal observation.

### Ecological niche modeling

We used ENMs to examine the potential range shifts of *R. crenulata* in response to glacial climatic oscillations. Locations of *R. crunulata* were obtained from field collections of the authors and from herbarium records online (e.g., Chinese Virtual Herbarium, National Specimen Information Infrastructure, and Global Biodiversity Information Facility). Data from online databases were checked to exclude misidentification. A total of 88 spatially unique localities were used for analysis. MAXENT v. 3.3.3e [50] was used to model the ecological niches of *R. crenulata*. Twenty replicates were employed, from which 80% of the distribution coordinates were used for training and 20% for testing. Environmental layers of 19 bioclimatic variables (Additional file 1: Table S1) for the Last Glacial Maximum (LGM), Last Interglacial (LIG), and the current time and the near future (year 2080) were downloaded from the WorldClim dataset at a spatial resolution of 2.5 arc-minutes and these were employed for the modeling [51]. To exclude highly correlated climate variables, pairwise correlations were examined among the 19 variables within the distribution area of *R. crenulata*. The nine variables (Additional file 1: Table S1) with pairwise Pearson correlation coefficients below 0.7 were used. Area under the “receiver operating characteristic (ROC) curve” (AUC) [52, 53] values were used to evaluate the accuracy of each model prediction. The threshold for good performance was set to 0.7 [54]. DIVA-GIS v. 7.5 [55] was used to draw the range of suitable distributions.

## Results

### Plastid DNA sequence variation and distribution

**Fig. 2:**
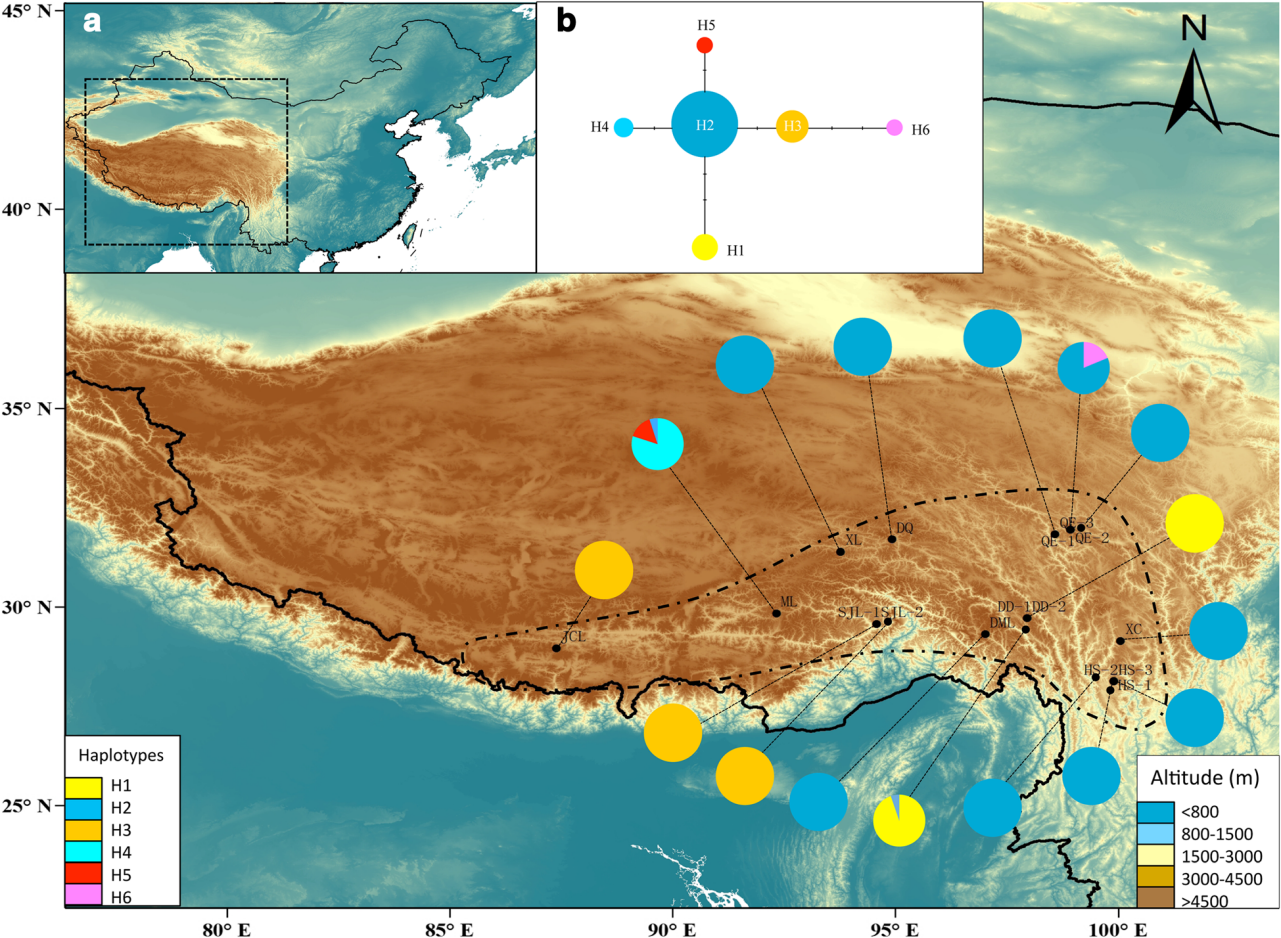
Map of the geographical distribution, and the phylogenetic network of cpDNA haplotypes of *R. crenulata*. **a** A map showing the sites of sampled populations and the geographical distribution of haplotypes. Pie charts show the proportion of haplotypes within each population. Dashed line on the map indicates the distribution area of *R. crenulata*. **b** NETWORK-derived genealogic relationships of cpDNA haplotypes. Different colors represent different haplotypes

The total alignment length of the two plastid DNA sequences was 1531 bp. In combination, we revealed six haplotypes determined by seven nucleotide substitutions. Of the 16 populations sampled, 13 were fixed for a single haplotype, and the remaining three were polymorphic (Table 1 & Additional file 2: Table S2; Fig. 2). Population ML harbored three haplotypes, which was the most of any of the populations. Three haplotypes (H4, H5 and H6) occurred only in one population each, while H2 was shared by 12 of the 16 populations. At species level, haplotype diversity was *h* = 0.554. Considering the populations, the highest haplotype diversity was detected in the ML population, with a value of 0.353 (Table 1). Nucleotide diversity was *pi* = 0.00063 at the overall scale, and the highest value was found in population QE-3, with 0.00068 (Table 1). Total gene diversity (*H*_T_) was much higher than within-population gene diversity (*H*_S_) (0.598 and 0.049, respectively, Table 2).

**Table 1:** Locations of populations of *R. crenulata* sampled, sample sizes (N), frequencies of cpDNA haplotypes and ITS sequences per population, and estimates of haplotype diversity and nucleotide diversity for chlorotypes and ribotypes within populations

Population ID	Province	Location	Alt. (m)	Lat. (N)	Long. (E)	N	Haplotypes nos.	*h* (SD)	*π* (SD)	ITS sequences nos.	*h* (SD)	*π* (SD)
DML	XZ	Demula Mt.	4900	29°18′36”	97°00′59”	20	H2(20)	0	0	R1(20)	0	0
DQ	XZ	Dingqing	4959	31°41′34”	94°55′31”	20	H2(20)	0	0	R1(20)	0	0
DD-1	XZ	Dongda Mt.	5080	29°42′46”	97°57′43”	19	H1(18) H2(1)	0.105 (0.092)	0.00014 (0.00012)	R1(19)	0	0
DD-2	XZ	Dongda Mt.	5080	29°42′46”	97°57′43”	14	H1(14)	0	0	R1(14)	0	0
JCL	XZ	Jiacuola Mt.	5505.5	28°57′28”	87°23′37”	16	H3(16)	0	0	R3(16)	0	0
ML	XZ	Mila Mt.	5225	29°50′02”	92°20′02”	20	H5(3) H4(16) H2(1)	0.353 (0.123)	0.00041 (0.00014)	R1(7) R3(2)R4(11)	0.595 (0.073)	0.00112 (0.00020)
SJL-1	XZ	Sejila Mt.	4728	29°33′62”	94°34′58”	12	H3(12)	0	0	R1(12)	0	0
SJL-2	XZ	Sejila Mt.	4728	29°33′62”	94°34′42”	10	H3(10)	0	0	R1(10)	0	0
XL	XZ	Xiala Mt.	5068	31°22′58”	93°46′32”	19	H2(19)	0	0	R1(9)R6 (10)	0.526 (0.040)	0.00083 (0.00006)
QE-1	SC	Que’er Mt.	4845	31°56′05”	98°55′43”	17	H2(17)	0	0	R1(11)R5 (6)	0.485 (0.079)	0.00077 (0.00012)
QE-2	SC	Que’er Mt.	4700	31°56′02”	98°56′02’	10	H2(10)	0	0	R1(10)	0	0
QE-3	SC	Que’er Mt.	4823	31°48′79”	98°34′78”	16	H6(3) H2(13)	0.325 (0.125)	0.00068 (0.00026)	R1(16)	0	0
XC	SC	Xiangcheng	4780	29°08′23”	100°03′24”	18	H2(18)	0	0	R1(6)R2(12)	0.471 (0.082)	0.00074 (0.00013)
HS-1	YN	Hongshan Mt.	4365	28°07′43”	99°53′51”	20	H2(20)	0	0	R1(18)R2(2)	0.189 (0.108)	0.00030 (0.00017)
HS-2	YN	Hongshan Mt.	4342	28°07′08”	99°54′02”	12	H2(12)	0	0	R1(12)	0	0
HS-3	YN	Hongshan Mt.	4342	28°07′08”	99°54′02”	10	H2(10)	0	0	R1(10)	0	0

XZ Xizang Province, SC Sichuan Province, YN Yunnan Province

**Table 2:** Genetic diversity and genetic differentiation of 16 populations of *R. crenulata* at the species and group levels

	*H*_s_	*H*_T_	*G*_ST_	*N*_ST_	*p*
cpDNA					
All population	0.049 (0.0291)	0.598 (0.1160)	0.918 (0.0455)	0.883 (0.0703)	*p >* 0.05
Clade I	0.017 (0.0116)	0.397 (0.1532)	0.957 (0.0158)	0.964 (0.0161)	*p* > 0.05
Clade II	–	–	–	–	–
ITS					
All population	0.142 (0.0578)	0.375 (0.1208)	0.622 (0.1170)	0.635 (0.1145)	*p >* 0.05
Clade A	0.068 (0.0464)	0.239 (0.1201)	0.718 (0.1641)	0.698 (0.1768)	*p >* 0.05

### NrDNA ITS variation and ribotype distribution

**Fig. 3:**
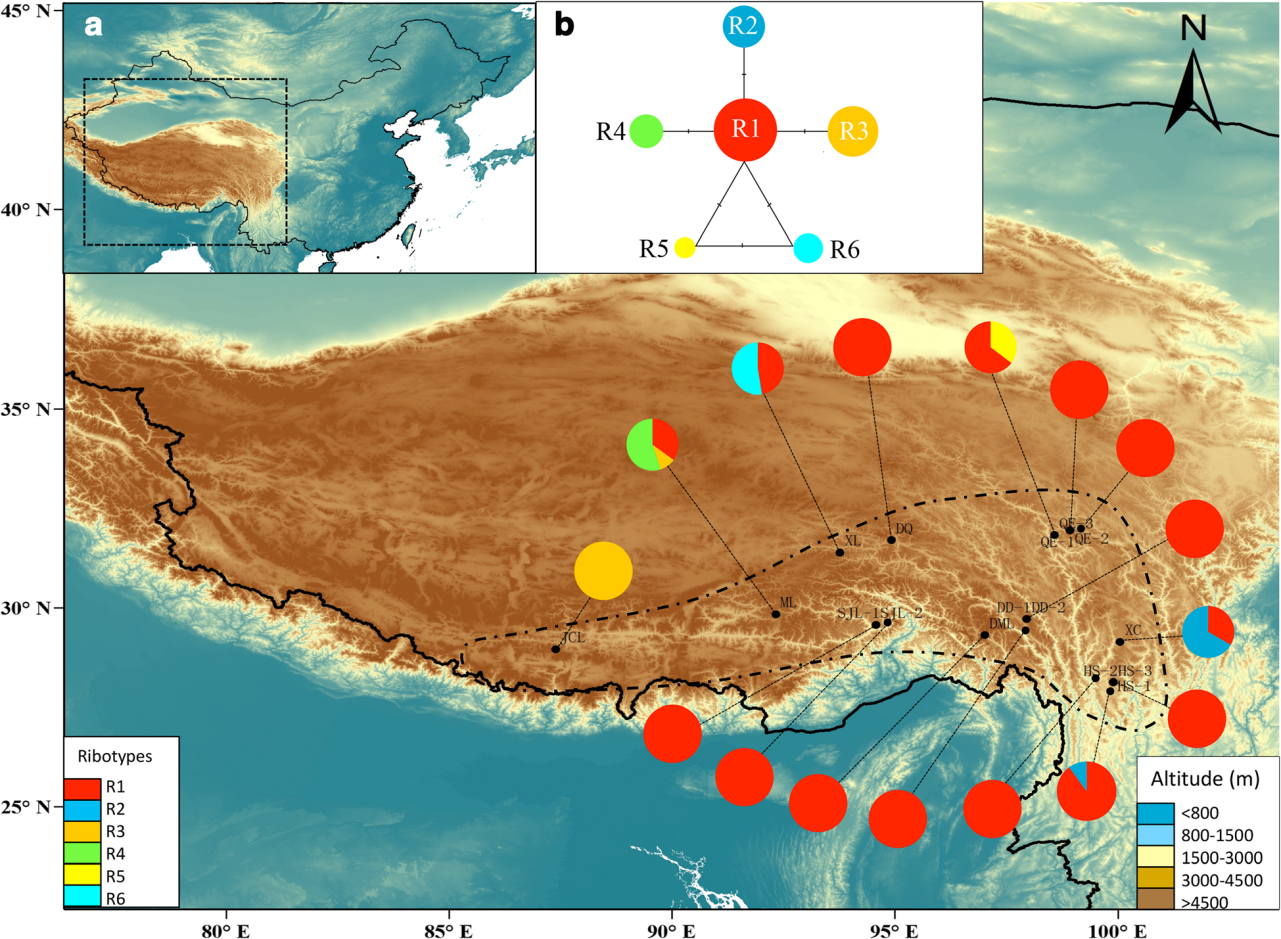
Map of the geographic distribution, and the phylogenetic network of ITS ribotypes of *R. crenulata*. **a** A map showing the sites of sampled populations and the geographic distribution of ITS ribotypes. Pie charts show the proportion of ribotypes within each population. Dashed line on the map indicates the distribution area of *R. crenulata*. **b** NETWORK-derived genealogic relationships of ITS ribotypes. Different colors represent different ribotypes

The total length of aligned ITS sequences was 606 bp, with a length variation from 604 to 606 bp. We detected six different ITS sequences (ribotypes) in all 253 individuals, which were determined by four substitutions. Among the six ribotypes, three occurred in only one population. R1 was the most widespread ribotype, occurring in 15 of the 16 populations we sampled (Table 1 & Additional file 3: Table S3; Fig. 3). Ribotype diversity was *h* = 0.401 at the species level. As with the cpDNA data, population ML had the highest *h* values (Table 1). The total nucleotide diversity was estimated to be 0.0007, and considering the populations, the highest was detected in the ML population (Table 1). Within-population gene diversity (*H*_S_) was also much lower than total gene diversity (*H*_T_) (0.142 and 0.375, respectively; Table 2) as demonstrated in the cpDNA data. Strikingly, 11 out of the 16 sampled populations only harbored a single ribotype.

### Phylogenetic relationships and lineage divergence

**Fig. 4:**
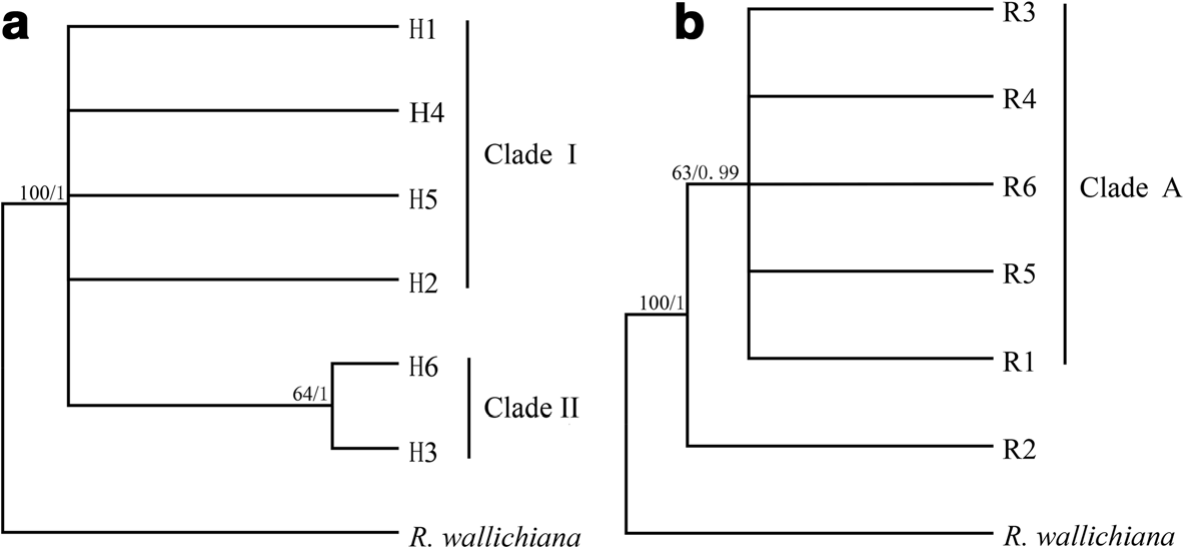
Phylogenetic relationships between haplotypes and ribotypes. **a** The Bayesian topology of the six haplotypes detected in *R. crenulata*. **b** The Bayesian topology of the six ITS ribotypes detected in *R. crenulata*. Numbers above the branches are MP bootstrap support values (left) and Bayesian posterior probability (right)

For both data sets, MP and Bayesian inference yielded largely congruent tree topologies, thus we only show the Bayesian tree with the MP bootstrap value marked on each branch. The phylogenetic relationship between haplotypes is shown in Fig. 4a. H3 and H6 comprised a moderately-supported clade (Fig. 4a), and together with four other haplotypes formed a polytomy. The haplotype network (Fig. 2b) revealed a consistent relationship between detected haplotypes within the phylogenetic tree (Fig. 4a),

Phylogenetic relationships for ITS ribotypes are shown in Fig. 4b. Ribotype 2 and other ribotypes diverged first, while the remaining five ribotypes formed a clade (BP = 63, PP = 0.99). The network relationships of ribotypes are shown in Fig. 3b, which is consistent with the phylogenetic trees (Fig. 3a), but exhibits a reticulate relationship.

**Fig. 5:**
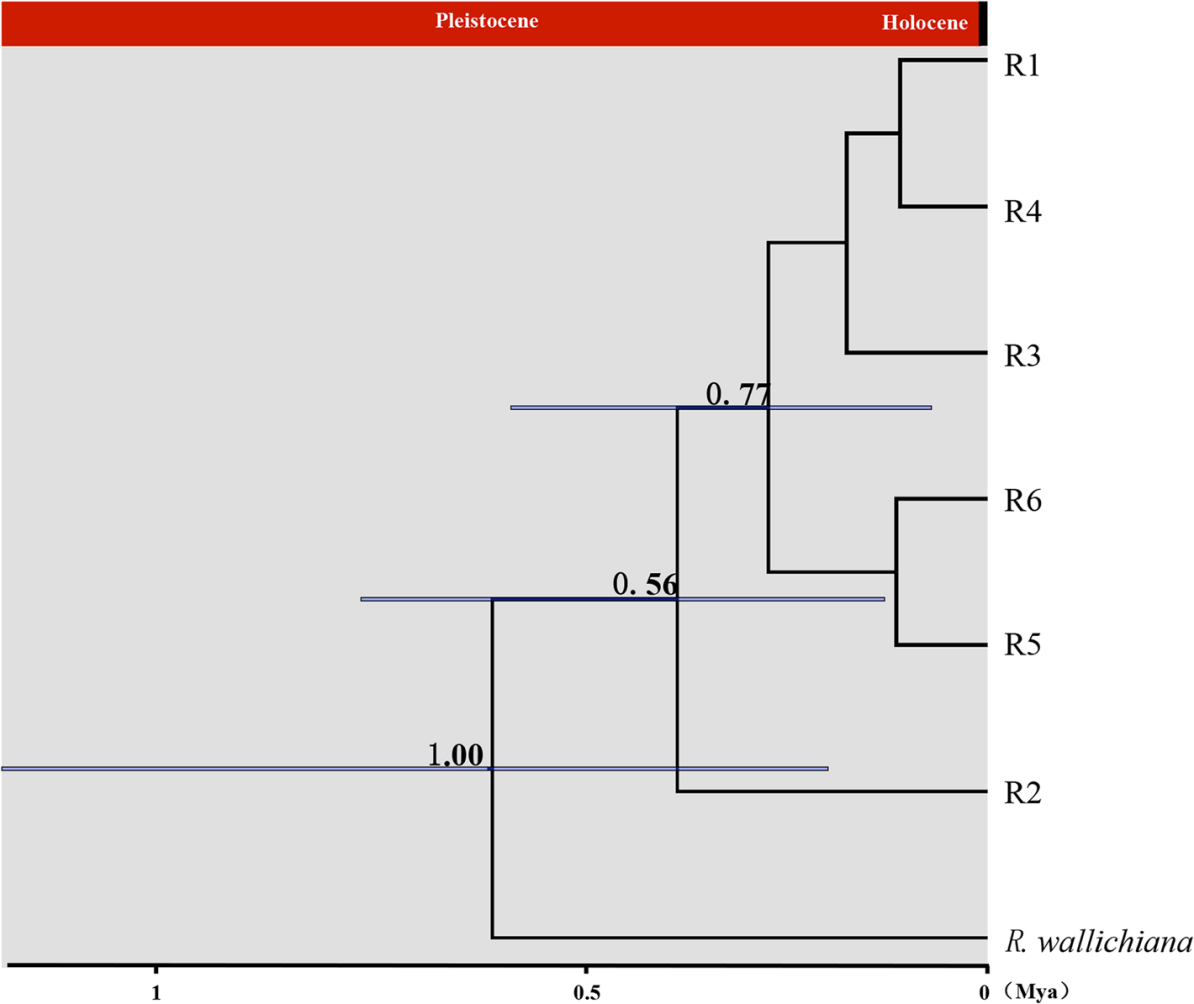
Divergence time of haplotypes of *R. crenulata* and the outgroup based on ITS ribotypes estimated with BEAST. Gray bars indicate 95% highest posterior density intervals

The dating analysis based on the ITS data showed that *R. crenulata* diverged from its closest relatives 0.65 Mya (95% HPD: 0.20–1.20 Mya), i.e. in the Pleistocene (Fig. 5). Further divergence of R2 and the other five ribotypes occurred 0.41 Mya (95% HPD: 0.13–0.76 Mya). Dating based on the cpDNA data set was consistent with that based on ITS data but with earlier dates: the divergence of *R. crenulata* and *R. wallichiana* occurred 1.25 Mya (95% HPD: 0.44–2.22 Mya), still in the Pleistocene (Additional file 4: Figure S1) and further divergence between H3, H6 and other haplotypes occurred 0.82 Mya (95% HPD: 0.29–1.44 Mya).

### Population structure

The *G*_ST_ values (0.918 and 0.622 for cpDNA and ITS data, respectively) (Table 2) showed high differentiation between *R. crenulata* populations. However, the permutation tests for both cpDNA and ITS data sets showed that *N*_ST_ was not significantly higher than *G*_ST_ (Table 2). In the SAMOVA analysis for the cpDNA data set, the *F*_CT_ value reached a plateau with *K* = 4 (Additional file 5: Figure S2). Population ML formed a group on its own, and DD-1 and DD-2 formed another group. The remaining populations were separated into two groups: JCL, SJL-1 and SJL-2 were in one group and the others in another group. For the ITS data set, *K* = 2 resulted in the maximum value, thus two groups were detected. JCL was an independent group, and the remaining populations formed another group. AMOVA analysis showed that for the cpDNA data set, 93.91% of the total variations occurred among groups according to SAMOVA. For the ITS data set, variations among groups also accounted for 69.72% of the total variation (Table 3). We found no pattern of geographic isolation revealed by the Mantel test based on both data sets.

**Table 3:** Analysis of molecular variance (amova) of chlorotypes and ITS ribotypes for *R. crenulata* populations

Source of variation	cpDNA					ITS				
	*df*	SS	VC	PV(%)	*F statistic*	*df*	SS	VC	PV(%)	*F statistic*
Among groups	3	62.590	0.45363 Va	93.91	*F*_SC_ = 0.03442*	1	12.450	0.37147 Va	69.72	*F*_SC_ = 0.47511*
Among populations	12	0.529	0.00101Vb	0.21	*F*_ST_ = 0.94117 (0.003)	14	18.066	0.07665 Vb	14.39	*F*_ST_ = 0.84106*
Within populations	237	6.735	0.02842 Vc	5.88	*F*_CT_ = 0.93907*	237	20.069	0.08468Vc	15.89	*F*_CT_ = 0.69720 (0.06647)
Total	252	69.854	0.48306			252	50.585	0.53279		

*df* degrees of freedom, *SS* sum of squares, *VC* variance components, *PV* percentage of variation. *F*_SC_, correlation withinpopulations relative togroup; *F*_ST_, correlation within populations relative to total; *F*_CT_, correlation within groups relative to total. *, *p* < 0.001, 1000 permutations. *F*_ST_ = 0.94117 (*p* = 0.003). *F*_CT_ = 0.6972 (*p* = 0.06647)

### Demographic analyses

Under the spatial population expansion model, a unimodel mismatch distribution was detected including all haplotypes (Additional file 6: Figure S3). We also found that observed variance (SSD) and the raggedness index were not significantly different from the expected values (Table 4). In addition, a negative value for Tajima’s *D* was calculated (Table 4). Thus, all evidence suggested that *R. crenulata* underwent a rapid expansion, the time of which was dated to around 0.31 Mya (Table 4). However, we failed to detect population expansion signals in the individual clades shown in Fig. 4a.

**Table 4:** Results of the mismatch distribution analysis and neutrality tests of *R. crenulata* and the two multiple-haplotype cpDNA clades

Haplotype group	T (Mya)	Tau	SSD	*p*-value	Raggedness index	*p*-value	Tajima’s *D*	*p*-value	*F*_S_	*p*-value
Clade I Clade II Total	–	0.000	0.231	0.000	0.270	0.960	−0.023	0.497	0.846	0.690
	–	3.000	0.026	0.130	0.780	0.680	−0.760	0.206	0.746	0.474
	0.31	1.488	0.001	0.860	0.533	0.880	−0.432	0.304	0.039	0.552

### Species distribution models for *R. crenulata*

**Fig. 6:**
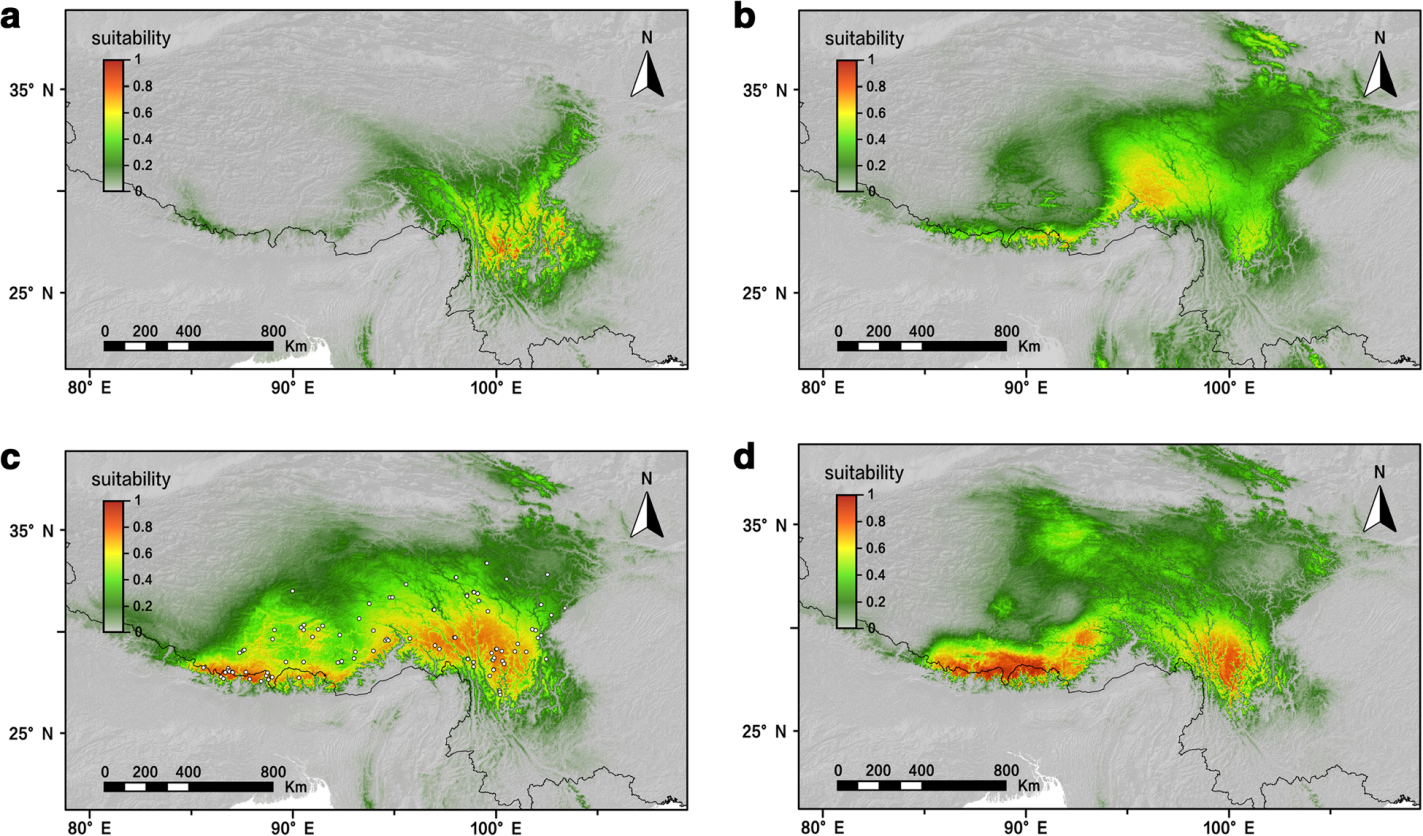
Distribution dynamics of *R. crenulata* during the LIG, the LGM, the present day, and in the future (2080) based on species distribution modeling using MAXENT. Predicted distributions are shown for **a** the LIG model, **b** the LGM model, **c** the present model, and **d** the 2080 model. White circles represent recorded locations used in the modelling. LGM, last glacial maximum; LIG, last interglacial

ENMs performed with high predictive ability, with AUC > 0.98 and standard deviation [SD] < 0.01 in each model. The predicted potential distributions of *R. crenulata* during the LIG, LGM, present day, and in the future are shown in Fig. 6. Our current sampling area covers most of the current potential distribution area of *R. crenulata* (Fig. 1c). In both the LIG and LGM models, the distribution area was smaller and also more fragmented than at present. Interestingly, the LIG model showed a more significant contraction to the Hengduan Mountain area (Fig. 6a). Focusing on higher habitat suitability (> 0.5), the existence of suitable habitats on the QTP was predicted in the LGM model (Fig. 6b), but not in the LIG model. We also modeled the potential distribution of *R. crenulata* under future climate change scenarios (year 2080) (Fig. 6d). We found that in 2080, *R. crenulata* will expand into the Himalaya area slightly, but will contract its distribution in the Hengduan Mountains compared to the present.

## Discussion

### Phylogeographic pattern of *R. crenulata*

Many authors have investigated how the Quaternary climate oscillations have shaped the distributions patterns of plants on the QTP and its adjacent areas [15–18, 39, 56–58]. Some species appear to comply with the massif de refuge hypothesis [12]: *Juniperus przewalskii* [39] and *Picea crassifolia* [56] showed reduced genetic diversity or even single haplotypes on the QTP platform but high levels of genetic diversity and private haplotypes in the Hengduan Mountains. On the other hand, refugia existed both on the plateau and in the Hengduan Mountains for *Potentilla glabra* [15] and *Aconitum gymnandrum* [16]. In addition, the *Juniperus tibetica* complex [17] and *Hippophae tibetana* [18] provide evidence that “microrefugia” existed across the distributional range.

Our results represent an interesting case: neither the nunataks nor the massif de refuge hypotheses can be rejected for *R. crenulata*, which is also the case for *Potentilla glabra* [15] and *Aconitum gymnandrum* [16]. Glacial refugia are characterized by high levels of genetic diversity and the presence of private haplotypes [59]. In the case of *R. crenulata*, both cpDNA and ITS data sets showed high genetic diversity and private haplotypes in population ML (Figs. 2 and 3; Table 1), which is on the plateau, and in QE and HS populations (Figs. 2 and 3; Table 1), which are located in the Hengduan Mountains. The ecological modeling results also showed that, during the LGM, there were suitable habitats for *R. crenulata* both on the QTP and in the Hengduan Mountains (Fig. 6b). These results indicate that glacial refugia exited both on the QTP platform and in the Hengduan Mountains.

In previous studies, it has been suggested that the south-eastern edge of the QTP and the Hengduan Mountians represented important refugia for the QTP species [16, 23, 56]. In the cpDNA data, populations located in this area, such as DD-1 and QE-3 had high genetic diversity and haplotype diversity (Table 1). In addition, population QE-3 harbored a private haplotype H6 (Fig. 2). A similar pattern can be seen in the ITS data set: population QE-1, XC and HS-1 had high genetic diversity and high haplotype diversity. Population QE-1 harbored private ribotype R5. Thus, our results confirmed that the south-eastern edge of the QTP and the Hengduan Mountains played an important role in hosting the genetic diversity of QTP plant species. Another population with high genetic diversity and haplotype uniqueness is ML, which is on the QTP. The refugium status was supported by both cpDNA and the ITS datasets. Interestingly, a previous study that focused on another *Rhodiola* species, *R. alsia*, also revealed that this area was a potential refugium for the species. Similarly, studies on *Potentilla glabra* [15] and *Aconitum gymnandrum* [16] also revealed in situ refugia on the QTP. *Rhodiola crenulata* often grows on schist on mountain slopes, rocky places and rock crevices at 4300–5600 m in elevation [19]. It is reasonable to suppose that it would have survived in situ on the QTP on nunataks that were ice free.

Our AMOVA analysis results suggest that 93.9% and 69.7% of the genetic diversity was found among groups defined by the SAMOVA analysis for the cpDNA and ITS data, respectively. It is generally expected that such genetic differentiation should be coupled with a distinct phylogeographic structure [60]. However, we found that neither cpDNA nor ITS datasets showed significantly higher *N*_ST_ than *G*_ST_ (Table 2), which indicated no phylogeographic structure across the distribution area of *R. crenulata*. We note that three of the six haplotypes (50%) and four out of the six ribotypes (67%) are endemic to one population. Furthermore, only one haplotype and one ribotype were widespread across the distribution range (Figs. 2 and 3). A small proportion of the widespread haplotype or ribotype and a larger proportion of endemic ones may cause the absence of phylogeographic structure in *R. crenulata*. Notably, H2 appeared in 12 of the 16 populations sampled, and R1 in 15 of the 16 populations. This pattern may have been the result of recent range expansion from glacial refugia. Mismatch distribution analysis based on the cpDNA dataset showed that all populations of *R. crenulata* as a whole experienced a range expansion, as indicated by negative Tajima’s *D* and a unimodal mismatch distribution (Table 4; Additional file 5: Figure S2). This expansion event was dated to 0.31 Mya, at the time of the interglacial between the Naynayxungla Glaciation (0.5–0.72 Mya) and the Guxiang Glaciation (0.13–0.3 Mya) [61]. The wide distribution of H2 and R1 may have been caused by this expansion event.

We also found a very low genetic diversity compared to congeneric species [23, 57] and other species in the same region [13–16, 39]. According to our results, this extremely low genetic diversity may be due to the species’ recent origin, as well as low within population diversity. In our field study, populations of *R. crenulata* are narrowly confined to mountain peaks, with a limited population size. This may be the reason for the low within population genetic diversity. Another possibility is that “Orbitally Forced Range Dynamics” – ORD [62, 63] have resulted in the current pattern of limited haplotype diversity. As *R. crenulata* is confined to glacial relic rocks, the Milankovitch climate oscillations may have repeatedly wiped out new mutations, as individuals carrying these mutations often went extinct during these oscillations.

### Implications for quaternary speciation

Although the role that the Pleistocene played in shaping intraspecific genetic diversity has been examined by a series of studies [5, 6, 64, 65], it is still debated whether speciation could have been triggered by the Pleistocene climate oscillations [66]. Palaeontological evidence has shown that most species remained unchanged throughout the Pleistocene [66]. However, an increasing number of studies reported speciation in the Quaternary [67–69, 70] and, indeed, the role of Quaternary speciation has attracted attention since Darwin [66]. Our dating analysis based on both cpDNA and ITS data sets clearly demonstrated that divergence between *R. crenulata* and its closest relative *R. wallichiana* occurred about 0.65 Mya, during the Naynayxungla glaciation on the QTP [61]. Although absolute dating of molecular phylogenies using the molecular clock method needs to be treated cautiously, our results suggest that the speciation of *R. crenulata* took place during the Pleistocene. The cpDNA data also suggested the divergence between *R. crenulata* and *R. wallichiana* be in the Pleistocene (Additional file 4: Figure S1). The evidence is in accordance with the fact that *R. crenulata* only grows on glacial relics, i.e. schist on mountain slopes, rocky places and rock crevices at 4300–5600 m in elevation. There were no glacial relics before the Pleistocene, so there were no suitable habitats for *R. crenulata*. It is reasonable to hypothesize that by adapting rapidly to glacial relics, *R. crenulata* diverged from its closest relative *R. wallichiana*, as the latter often grows at the moist margin of forests. Further studies based on extensive low copy nuclear markers and coalescence calculations are needed to elucidate the detailed evolutionary history of the two species.

### Conservation and management in the future

The roots and rhizomes of *R. crenulata* have been included in the Pharmacopoeia of China [71] as the authentic Rhodiolae Crenulatae Radix et Rhizoma, where they are listed as enhancing inner spiritual power, concentration and physical endurance. Since the 1980s, the accelerated and uncontrolled use of *R. crenulata* in China has severely reduced its population. Population genetic studies with inter-simple sequence repeat markers have demonstrated low genetic diversity of this species [72]. To preserve this threatened and endemic species, conservation programs should be planned and carried out. An ideal conservation program should involve all known populations, and populations that harbor higher genetic diversity, higher heterozygosity and unique haplotypes should be given priority. As our AMOVA analysis showed that most genetic variation exists among groups, conservation programs should be undertaken in each of the four groups. In particular, populations on Mila Mt. and Queer Mt. should be given the highest priority, because populations there had the highest genetic diversity and harbored multiple unique haplotypes (Figs. 2 and 3).

Another suggestion came from our ENMs analysis, which is helpful when developing conservation and management strategies because it predicts potential changes in geographic distribution under future climate change [73, 74]. It is reasonable to hypothesize that in a warming climate, mountain species will migrate toward higher altitudes [75]. As *R. crenulata* already grows at very high altitudes, as the climate becomes warmer, its suitable habitats will shrink. This hypothesis was confirmed by our ENMs (Fig. 6d): compared to the current distribution, the distribution of *R. crenulata* would shrink into two major areas, one in the Hengduan Mountains and the other one on the QTP. In addition, our ENMs suggested that based on the global warming model (Fig. 6d), populations from lower altitudes will experience lower habitat suitability (< 0.5). From the perspective of conservation management, populations that face a higher extinction risk, which are in low altitudes, should be given priority for protection (e.g., XL, DQ). Areas around Mila Mt. and Queer Mt. all possess high habitat suitability (> 0.5), therefore should be listed as the core area for conservation of this species; this is supported by both our genetic data and ENMs.

## Conclusion

We used two molecular markers and ecological niche modeling to elucidate the glacial history of *R. crenulata*, a perennial herb almost exclusively restricted to rock crevices on mountain peaks. Our results detected a strikingly recent divergence of *R. crenulata* from its closest relative, which was dated to around 0.65 Mya, during the Naynayxungla glaciation on the QTP. This result suggested that Quaternary climatic oscillations may have promoted plant speciation. In addition, we found that refugia existed in both the Hengduan Mountain area and on the QTP platform during the LGM, demonstrating a scenario that both the nunataks and the massif de refuge hypotheses apply to *R. crenulata*. Our study adds to a growing body of evidence suggesting that the QTP plant lineages exhibited diverse reactions to the Quaternary climatic oscillations.

## Additional files

Additional file 1: **Table S1.** Bioclimatic variables (BIO1 to BIO19) from WorldClim [51]. Variables marked with an asterisk (*) were used for the climatic niche models for *Rhodiola crenulata*. (DOCX 67 kb)

Additional file 2: **Table S2.** Haplotype composition of 16 sampled populations of *Rhodiola crenulata* based on the cpDNA data set. (DOCX 16 kb)

Additional file 3: **Table S3.** Haplotype composition of 16 sampled populations of *Rhodiola crenulata* based on the ITS data set. (DOCX 15 kb)

Additional file 4: **Figure S1.** Divergence time of *R. crenulata* and its closest relative based on the plastid DNA haplotypes estimated with BEAST. Gray bars indicates 95% highest posterior density intervals. (TIF 93 kb)

Additional file 5: **Figure S2.** Correlation between the *F* statistics and grouping number (*K* = 2–10) from the SAMOVA results. (a) results based on cpDNA haplotypes; (b) results based on ITS ribotypes. (TIF 887 kb)

Additional file 6: **Figure S3.** Historical demography for overall populations and in each clade based on the plastid DNA dataset. Clade I and clade II correspond to the groups in the Bayesian phylogenetic tree in Fig. 4a. Mismatch distribution showing histogram of observed mismatch frequencies and best-fit curve of the sudden expansion model. (TIF 2056 kb)

## Abbreviations

AIC: Akaike Information Criterion
AMOVA: Analysis of Molecular Variance
AUC: Area Under the Curve
BI: Bayesian Inference
ENMs: Ecological Niche Models
*HRag*: Raggedness index of Harpending
LGM: Last Glacial Maximum
LIG: Last Interglacial
PCR: Polymerase Chain Reaction
PP: Posterior Probabilities
QTP: Qinghai-Tibetan Plateau
ROC: Receiver Operating Characteristic
SAMOVA: Spatial Analysis of Molecular Variance
SD: Standard Deviation
SSD: Sum of Squared Deviations
