# Supplementary material for: Nunataks or massif de refuge? A phylogeographic study of Rhodiola crenulata (Crassulaceae) on the world’s highest sky islands
Source: BMC Evol Biol. 2018 Oct 16;18:154. doi: 10.1186/s12862-018-1270-6 (PMC6192188; doi:10.1186/s12862-018-1270-6)
Supplement: Supplementary file 1 — Table S1. Bioclimatic variables (BIO1 to BIO19) from WorldClim [51]. Variables marked with an asterisk (*) were used for the climatic niche models for Rhodiola crenulata. (DOCX 67 kb) [file 12862_2018_1270_MOESM1_ESM.docx]

**Table S1.** Bioclimatic variables (named BIO1 to BIO19) from WorldClim (Hijmans et al., 2005). Variables marked with an asterisk (*) were used for the climatic niche models of *R. crenulata*.

| Bioclimatic variables |  |
| --- | --- |
| BIO1 | Mean annual temperature |
| BIO2* | Mean diurnal range (mean of monthly (max temp—min temp)) |
| BIO3* | Isothermality (BIO2/BIO7) |
| BIO4 | Temperature seasonality (standard deviation) |
| BIO5 | Max temperature of warmest month |
| BIO6* | Min temperature of coldest month |
| BIO7* | Temperature annual range (BIO5–BIO6) |
| BIO8 | Mean temperature of wettest quarter |
| BIO9 | Mean temperature of driest quarter |
| BIO10 | Mean temperature of warmest quarter |
| BIO11 | Mean temperature of coldest quarter |
| BIO12 | Annual precipitation |
| BIO13* | Precipitation of wettest month |
| BIO14* | Precipitation of driest month |
| BIO15* | Precipitation seasonality (coefficient of variation) |
| BIO16 | Precipitation of wettest quarter |
| BIO17 | Precipitation of driest quarter |
| BIO18* | Precipitation of warmest quarter |
| BIO19* | Precipitation of coldest quarter |
