# Supplementary material for: Nunataks or massif de refuge? A phylogeographic study of Rhodiola crenulata (Crassulaceae) on the world’s highest sky islands
Source: BMC Evol Biol. 2018 Oct 16;18:154. doi: 10.1186/s12862-018-1270-6 (PMC6192188; doi:10.1186/s12862-018-1270-6)
Supplement: Supplementary file 2 — Table S2. Haplotype composition of 16 sampled populations of Rhodiola crenulata based on the cpDNA data set. (DOCX 16 kb) [file 12862_2018_1270_MOESM2_ESM.docx]

**Table S2**. Haplotype composition of 16 sampled populations of *R. crenulata* based on the cpDNA data set

| **Population** | Haplotype composition | | | | | |
| --- | --- | --- | --- | --- | --- | --- |
|  | 1 | 2 | 3 | 4 | 5 | 6 |
| **DML** |  | 20 |  |  |  |  |
| **DQ** |  | 20 |  |  |  |  |
| **DD_1** | 18 | 1 |  |  |  |  |
| **DD_2** | 14 |  |  |  |  |  |
| **JCL** |  |  | 16 |  |  |  |
| **ML** |  | 1 |  | 16 | 3 |  |
| **SJL_1** |  |  | 12 |  |  |  |
| **SJL_2** |  |  | 10 |  |  |  |
| **XL** |  | 19 |  |  |  |  |
| **QE_1** |  | 17 |  |  |  |  |
| **QE_2** |  | 10 |  |  |  |  |
| **QE_3** |  | 13 |  |  |  | 3 |
| **XC** |  | 18 |  |  |  |  |
| **HS_1** |  | 20 |  |  |  |  |
| **HS_2** |  | 12 |  |  |  |  |
| **HS_3** |  | 10 |  |  |  |  |
