# Supplementary material for: Nunataks or massif de refuge? A phylogeographic study of Rhodiola crenulata (Crassulaceae) on the world’s highest sky islands
Source: BMC Evol Biol. 2018 Oct 16;18:154. doi: 10.1186/s12862-018-1270-6 (PMC6192188; doi:10.1186/s12862-018-1270-6)
Supplement: Supplementary file 3 — Table S3. Haplotype composition of 16 sampled populations of Rhodiola crenulata based on the ITS data set. (DOCX 15 kb) [file 12862_2018_1270_MOESM3_ESM.docx]

**Table S3.** Haplotype composition of 16 sampled populations of *R. crenulata* based on the ITS data set

| **Population** | Haplotype composition | | | | | |
| --- | --- | --- | --- | --- | --- | --- |
|  | 1 | 2 | 3 | 4 | 5 | 6 |
| **DML** | 20 |  |  |  |  |  |
| **DQ** | 20 |  |  |  |  |  |
| **DD_1** | 19 |  |  |  |  |  |
| **DD_2** | 14 |  |  |  |  |  |
| **JCL** |  |  | 16 |  |  |  |
| **ML** | 7 |  | 2 | 11 |  |  |
| **SJL_1** | 12 |  |  |  |  |  |
| **SJL_2** | 10 |  |  |  |  |  |
| **XL** | 9 |  |  |  |  | 10 |
| **QE_1** | 11 |  |  |  | 6 |  |
| **QE_2** | 10 |  |  |  |  |  |
| **QE_3** | 16 |  |  |  |  |  |
| **XC** | 6 | 12 |  |  |  |  |
| **HS_1** | 18 | 2 |  |  |  |  |
| **HS_2** | 12 |  |  |  |  |  |
| **HS_3** | 10 |  |  |  |  |  |
