# Supplementary figures and images for: Nunataks or massif de refuge? A phylogeographic study of Rhodiola crenulata (Crassulaceae) on the world’s highest sky islands
Source: BMC Evol Biol. 2018 Oct 16;18:154. doi: 10.1186/s12862-018-1270-6 (PMC6192188; doi:10.1186/s12862-018-1270-6)

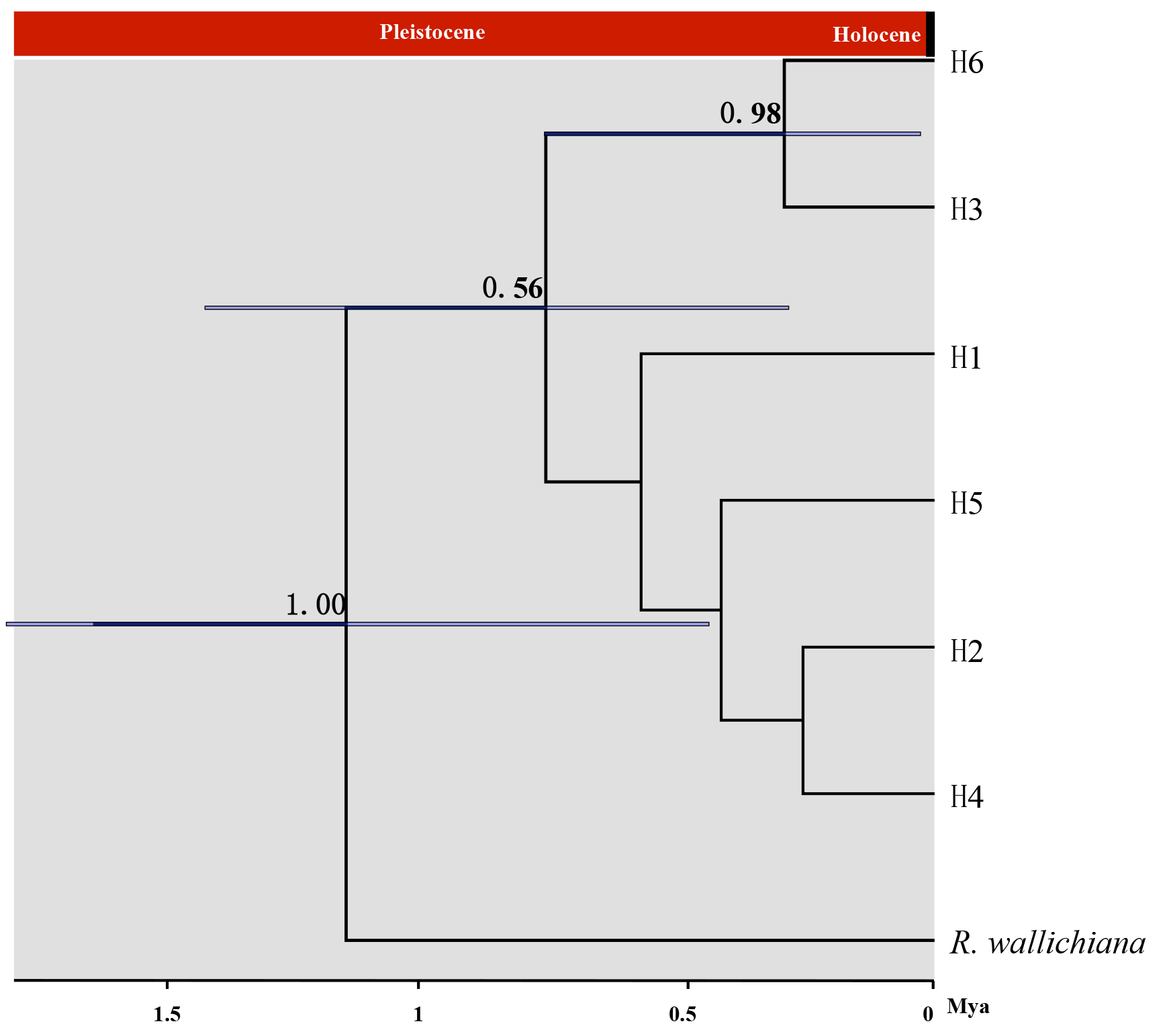

Supplement: Supplementary file 4 — Figure S1. Divergence time of R. crenulata and its closest relative based on the plastid DNA haplotypes estimated with BEAST. Gray bars indicates 95% highest posterior density intervals. (TIF 93 kb) [file 12862_2018_1270_MOESM4_ESM.tif]

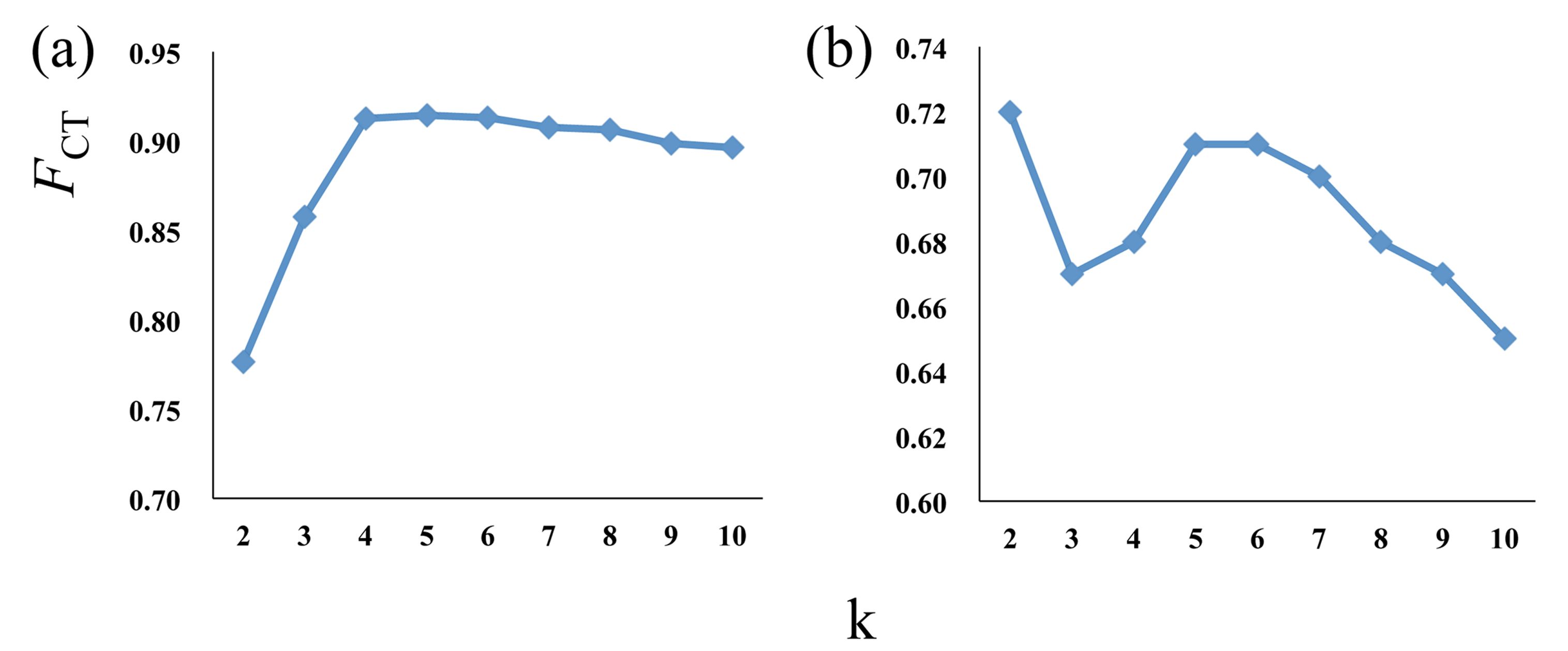

Supplement: Supplementary file 5 — Figure S2. Correlation between the F statistics and grouping number (K = 2–10) from the SAMOVA results. (a) results based on cpDNA haplotypes; (b) results based on ITS ribotypes. (TIF 887 kb) [file 12862_2018_1270_MOESM5_ESM.tif]

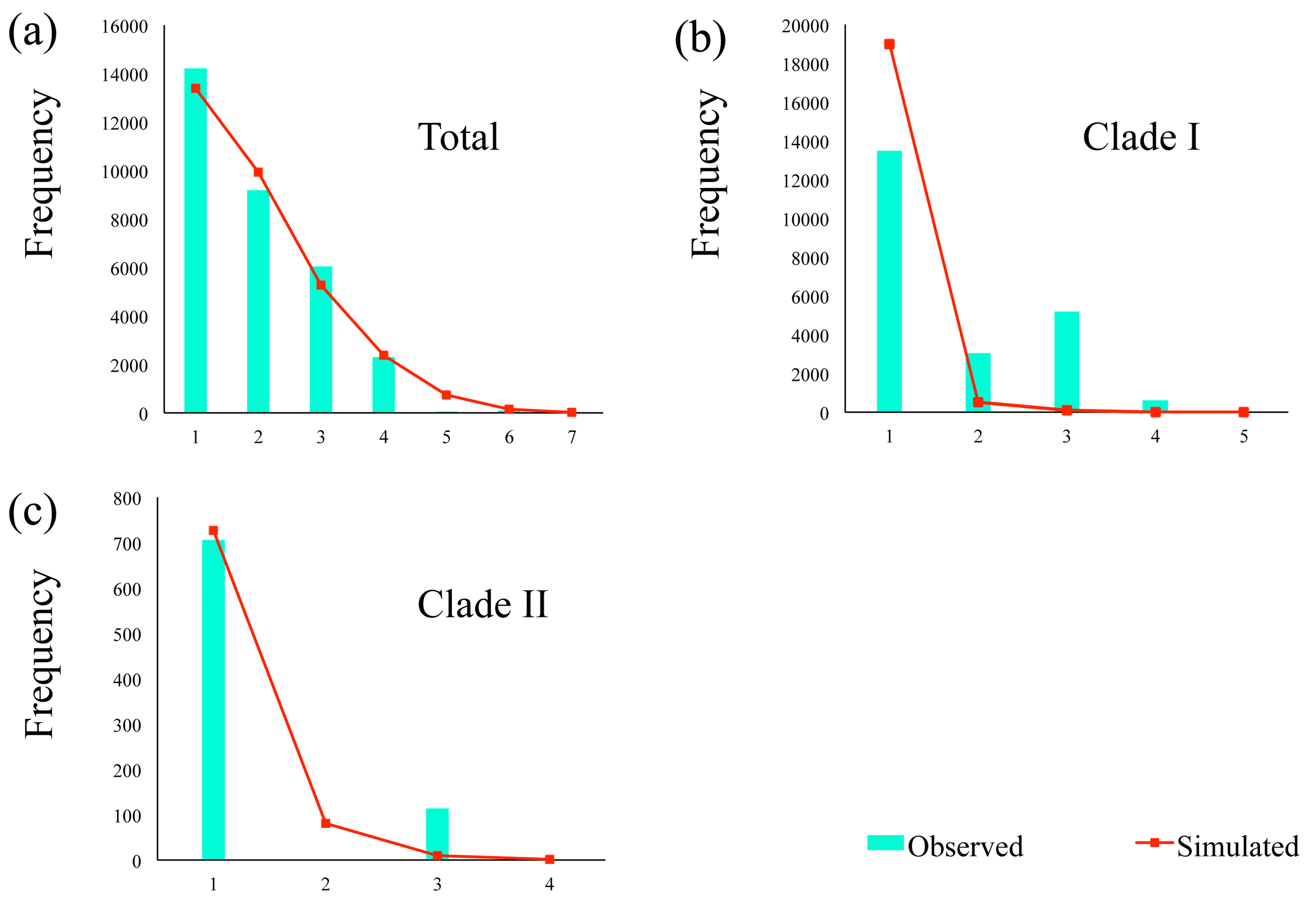

Supplement: Supplementary file 6 — Figure S3. Historical demography for overall populations and in each clade based on the plastid DNA dataset. Clade I and clade II correspond to the groups in the Bayesian phylogenetic tree in Fig. 4a. Mismatch distribution showing histogram of observed mismatch frequencies and best-fit curve of the sudden expansion model. (TIF 2056 kb) [file 12862_2018_1270_MOESM6_ESM.tif]
